# Research advances on the urinary microbiome in non-infectious urinary tract diseases: from community composition to clinical prospects

**DOI:** 10.3389/fcimb.2026.1728182

**Published:** 2026-03-03

**Authors:** Yalong Zhang, Hao Wang, Rui Yan, Kangyu Wang, Jiangwei Man, Li Yang

**Affiliations:** 1 Department of Urology, The Second Hospital & Clinical Medical School, Lanzhou University, Lanzhou, Gansu, China; 2 Gansu Province Clinical Research Center for Urinary System Disease, Lanzhou, Gansu, China

**Keywords:** biomarker discovery, metagenomic sequencing, microbiome-based therapy, non-infectious urological diseases, urinary microbiome

## Abstract

**Introduction:**

With the rapid development of 16S rRNA sequencing and metagenomic technologies, the traditional concept of sterile urine has been completely overturned, and a diverse urinary microbiome has been identified even in healthy individuals. Increasing evidence indicates that dysbiosis of the urinary microbiome is closely associated with the onset and progression of various non-infectious urological diseases.

**Methods:**

This review systematically summarizes recent advances in the role of the urinary microbiome in non-infectious urological diseases, including bladder cancer, benign prostatic hyperplasia, prostate cancer, nephrolithiasis, interstitial cystitis/bladder pain syndrome, and urinary incontinence, with a focus on microbial dysbiosis, pathogenic mechanisms, and clinical applications.

**Results:**

Studies have shown that alterations in the composition and diversity of the urinary microbiome are closely related to chronic inflammation, immune dysregulation, metabolic disturbances, and changes in the local microenvironment. These alterations may contribute to disease pathogenesis through mechanisms such as persistent low-grade inflammation, abnormal metabolic activity, and biofilm formation. In recent years, non-invasive detection based on urinary microbial profiles has shown promising potential in the early diagnosis of bladder and prostate cancers, with some machine learning models achieving diagnostic accuracies above 80 percent. Furthermore, the urinary microbiome may influence the efficacy of immunotherapy, offering new insights for personalized precision medicine.

**Conclusions:**

This review summarizes the mechanisms, research status, and clinical prospects of the urinary microbiome in non-infectious urological diseases, emphasizing the importance of methodological standardization and highlighting its potential applications in early screening, diagnostic stratification, and microbiome-targeted interventions.

## Introduction

Traditionally, it has long been believed that urine from healthy individuals is sterile when collected under aseptic conditions. However, with the advent of molecular biology techniques, particularly 16S rRNA gene sequencing and expanded quantitative urine culture, this dogma of “sterile urine” has been completely overturned (Aragón et al., 2018). Studies have demonstrated that even in the absence of clinical signs of infection, urine from healthy individuals contains a diverse microbial community, giving rise to the core concept of the “urinary microbiome” (Reasoner et al., 2025). The urinary microbiome refers to the entire assemblage of microorganisms inhabiting the urinary tract, including bacteria, viruses, and fungi. Its dynamic equilibrium is essential for maintaining urinary tract health, while its dysregulation may be closely associated with the development of various disease conditions (Pastuszka et al., 2025).

At present, research on the urinary microbiome primarily relies on three major methodological approaches: 16S rRNA gene sequencing, shotgun metagenomic sequencing, and enhanced quantitative urine culture (EQUC) (Han et al., 2020). The 16S rRNA gene–based approach amplifies conserved bacterial regions and provides a cost-effective approach for characterizing bacterial community composition (Regueira-Iglesias et al., 2023) However, as a DNA-based method, it cannot distinguish between viable and non-viable microorganisms, and therefore does not necessarily reflect active colonization or functional relevance. Shotgun metagenomic sequencing performs unbiased sequencing of all DNA within a sample, allowing for more accurate species identification and deeper exploration of functional gene profiles that may elucidate potential microbial mechanisms (Zhou et al., 2022). Nevertheless, similar to 16S sequencing, metagenomics primarily captures genomic material and cannot directly determine microbial viability. EQUC complements these sequencing-based methods by recovering viable, low-abundance, and fastidious organisms that are often underdetected by molecular techniques, thereby providing a more complete representation of the urobiome (Barnes et al., 2021). Integrating culture-based and sequencing-based methods therefore represents a more robust strategy for accurately characterizing urinary microbial ecosystems.

Despite these advances, methodological variability continues to pose challenges. Variations in sample collection, preservation, processing, and analytical pipelines have historically contributed to substantial heterogeneity across studies (Aggarwal et al., 2023). However, in recent years, two expert consensus statements have introduced comprehensive standardization frameworks covering specimen terminology, sampling protocols, storage conditions, metadata requirements, and bioinformatic reporting, marking significant progress toward reducing cross-study variability and improving reproducibility (Brubaker et al., 2021; Kachroo et al., 2021). Promoting methodological standardization has thus become a widely recognized prerequisite for further breakthroughs in the field. Although significant challenges persist, urinary microbiome research has opened a new perspective for understanding urinary tract health and disease. Accumulating evidence indicates that dysbiosis of the urinary microbiome is associated with the development and progression of a range of non-infectious urological disorders, including bladder cancer, interstitial cystitis/bladder pain syndrome, benign prostatic hyperplasia, prostate cancer, nephrolithiasis, and urinary incontinence (Jones-Freeman et al., 2021). However, compared with its well-established role in urinary tract infections (Saenz et al., 2024), the specific functions, underlying mechanisms, and clinical translational potential of the urinary microbiome in non-infectious diseases remain to be systematically elucidated.

Therefore, this review aims to systematically summarize the latest research progress on the urinary microbiome in major non-infectious urological diseases. We focus on characterizing the distinct microbial alterations observed under different disease contexts, exploring the potential molecular mechanisms underlying microbial dysbiosis and host interactions, and evaluating the prospects of the urinary microbiome as a novel diagnostic biomarker and therapeutic target. Ultimately, through the comprehensive integration of current knowledge, we seek to provide a theoretical framework and future outlook that may facilitate the translation of urinary microbiome research from basic science to clinical application, thereby advancing precision diagnosis and treatment of urological diseases. To provide an integrated overview of current evidence, representative studies on urinary microbiome alterations across major non-infectious urological diseases are summarized in **Table 1**.

**Table 1:** Summary of urinary microbiome alterations across major non-infectious urological diseases.

Disease	Sample type	Main methods	Key microbial features	Potential clinical relevance	Representative studies
BCa	Midstream urine; urine; tissue	16S rRNA; 2bRAD-M; ML	Enrichment of *Fusobacteria*, *Actinobacteria*, *Acinetobacter*, *Actinomyces*; increased diversity; sex-specific patterns	Auxiliary diagnosis; recurrence prediction; immunotherapy response; subtyping	Popovic 2018 (Bučević Popović et al., 2018); Chorbinska 2023 (Chorbińska et al., 2023); Hussein 2021 (Hussein et al., 2021); Sheng 2025 (Sheng et al., 2025); Chipollini 2020 (Chipollini et al., 2020); Wu 2018 (Wu et al., 2018); Bi 2019 (Bi et al., 2019); Pederzoli 2020 (Pederzoli et al., 2020); Sun 2023 (Sun et al., 2023); Zeng 2020 (Zeng et al., 2020); Chen 2022 (Chen et al., 2022)
PCa	Urine; tissue; feces	16S rRNA	*Propionibacterium acnes* and *Fusobacterium* enrichment; reduced diversity	Early screening; diagnostic subtyping	Alanee 2019 (Alanee et al., 2019); Ahn 2022 (Ahn et al., 2022); Shrestha 2018 (Shrestha et al., 2018); Cavarretta 2017 (Cavarretta et al., 2017)
RCC	First-void urine; urine	16S rRNA	Enrichment of *Gardnerella* and *Enterococcus*; altered richness	Screening; mechanistic insight	Ahn 2022 (Ahn et al., 2022); Cumpanas 2024 (Cumpanas, [[NoYear]])
BPH	Prostate tissue; urine; animal model	Metagenomics; 16S rRNA; metabolomics	Microbial imbalance; increased *Eubacterium* and *Defluviicoccus*; SCFA dysregulation	Inflammatory assessment; risk prediction; drug target screening	Oseni 2023 (Oseni et al., 2023); Bowie 2025 (Bowie et al., 2025); Mariotti 2024 (Mariotti et al., 2024); Li 2022 (Li et al., 2022)
Kidney stones	Urine; feces; stones; animal model	16S rRNA; metagenomics; multi-omics	Increased diversity; distinct stone-associated microbiota; altered metabolites	Recurrence risk; mechanism exploration; microbial therapy	Lemberger 2023 (Lemberger et al., 2023); Gao 2022 (Gao et al., 2022); Wan 2024 (Wan et al., 2024)
IC/BPS	Catheter urine; urine	16S rRNA; metabolomics; culture	Reduced *Lactobacillus*; dysbiosis; metabolic reprogramming	Diagnostic stratification; therapeutic monitoring	Abernethy 2017 (Abernethy et al., 2017); Zheng 2023 (Zheng et al., 2023); Nickel 2019 (Nickel et al., 2019); Chen 2025 (Chen et al., 2025)
UI	Catheter urine; urine	16S rRNA	*Gardnerella* enrichment; *Lactobacillus* decline; diversity changes	Risk prediction; symptom stratification	Pearce 2014 (Pearce et al., 2014); Komesu 2018 (Komesu et al., 2018); Carnes 2024 (Carnes et al., 2024); Karstens 2016 (Karstens et al., 2016)

Bca, Bladder cancer; ML, machine learning; Pca, Prostate cancer; RCC, Renal cell carcinoma; BPH, Benign prostatic hyperplasia; SCFA, short-chain fatty acids; IC/BPS, Interstitial cystitis/bladder pain syndrome; UI, Urinary incontinence.

## Fundamental concepts and detection techniques of the urinary microbiome

The urinary microbiome refers to the collective community of microorganisms that reside within the urinary tract. Its existence has been confirmed through multiple complementary techniques, including 16S rRNA gene sequencing, which detects bacterial DNA, and culture-based techniques such as EQUC. Additionally, RNA-based sequencing approaches such as total RNA sequencing offer improved inference of bacterial viability because RNA is rapidly degraded in nonviable cells (Perez-Carrasco et al., 2021). In healthy individuals, the urinary microbiome exhibits clear sex-specific characteristics. In males, the predominant phylum of the urinary microbiome is *Bacillota*, accounting for approximately 53 percent, followed by*Actinobacteriota*, *Fusobacteria*, and *Proteobacteria*. At the genus level, common taxa include *Lactobacillus complex*, *Corynebacterium*, and *Streptococcus* (Nelson et al., 2010). In females, the proportion of *Bacillota* is even higher, reaching about 65 percent, with relatively greater abundances of *Actinobacteriota* and *Bacteroidota*. The dominant genus is *Lactobacillus complex*, followed by *Prevotella* and *Gardnerella* (Siddiqui et al., 2011).

Overall, the female urinary microbiome exhibits higher diversity than that of males and is characterized by the predominance of Lactobacillus complex, which is likely influenced by the close ecological relationship between the urinary and vaginal microbiota. The urinary microbiome is not a static entity but a dynamic ecosystem that is influenced by multiple factors, including sex, age, hormonal status, and behavioral habits (Whiteside et al., 2015). For instance, in females, microbial diversity tends to decrease progressively with aging (Fouts et al., 2012; Lewis et al., 2013).

After confirming the existence of the urinary microbiome and elucidating its basic characteristics, the selection and optimization of reliable detection techniques have become crucial for advancing research in this field. Currently, studies mainly rely on two major methodological approaches: amplicon-based molecular sequencing and culture-based enhanced methods. Among them, 16S rRNA gene amplicon sequencing has become the preferred technique for characterizing bacterial community composition because of its relatively low cost and mature workflow (Heidrich et al., 2022). However, this technique depends on specific primers targeting conserved gene regions, and its taxonomic resolution is generally limited to the genus level. It also lacks the ability to effectively detect non-bacterial members. To address this, researchers are increasingly employing Internal Transcribed Spacer (ITS) sequencing to specifically characterize the urinary mycobiome. Furthermore, shotgun metagenomic sequencing has emerged as a critical tool for multi-domain analysis. By sequencing all DNA within a sample without target amplification, metagenomics enables the simultaneous detection of bacteria, fungi, and viruses, providing a comprehensive functional and taxonomic profile of the urinary ecosystem. This enables more accurate species identification and simultaneously reveals the functional gene profiles of microbial communities, providing important insights into potential mechanisms (Shi et al., 2022). Shotgun metagenomic sequencing has also been increasingly applied in urinary microbiome research across a range of urological conditions. Compared with 16S rRNA sequencing, shotgun-based profiling provides higher taxonomic resolution and enables the detection of fungi, viruses, and low-abundance bacterial species that are often missed by amplicon-based methods. Indeed, several multi-kingdom studies have reported the presence of diverse viral and fungal communities in urine, suggesting potential interactions between bacteria, viruses, fungi, and host immunity in the urinary tract (Singla et al., 2012; Javanmard et al., 2019; Robles et al., 2020). However, compared with bacterial profiling, investigations of the urinary virome and mycobiome remain limited, and their clinical relevance has yet to be fully elucidated.

Nevertheless, shotgun metagenomics has already demonstrated substantial value in characterizing disease-associated microbial functions and dysbiosis patterns in urological disorders. This approach has been used to delineate functional microbial pathways in bladder cancer (Knorr et al., 2024), to explore dysbiosis signatures in overactive bladder and interstitial cystitis (Bersanelli et al., 2019), and to identify virulence- and metabolism-related gene profiles associated with recurrent urinary tract symptoms (Lewis et al., 2025). These applications highlight the expanding utility of shotgun metagenomics in revealing both the taxonomic architecture and functional potential of the urobiome. Nevertheless, this approach requires higher DNA quality and input quantity, is more expensive, and remains technically challenging for low-biomass urine samples. Recent urinary microbiome studies have also emphasized the need for urine-specific contamination control strategies (Brubaker et al., 2021), including parallel processing of multiple types of negative controls, quantification of microbial biomass to guide filtering thresholds, and statistical approaches such as prevalence-based or frequency-based contaminant identification (Hilt et al., 2014). Several analyses have further demonstrated that inadequate removal of reagent or environmental contaminants can substantially bias taxonomic profiles and downstream association testing in low-biomass urine samples. Incorporating these methods has therefore become essential for improving the robustness and interpretability of urinary microbiome data (Davis et al., 2018; Karstens et al., 2019; Zinter et al., 2019).

It is important to note that molecular sequencing cannot distinguish between viable and non-viable microorganisms. In such cases, EQUC and other improved cultivation techniques serve as valuable complementary methods. By employing multiple media types, extending incubation times, and simulating anaerobic conditions, these approaches have successfully recovered previously “unculturable” microorganisms, providing viable isolates for subsequent functional studies (Thapaliya et al., 2020). Because the microbial DNA content in urine is extremely low, contamination from sampling procedures, laboratory reagents, or environmental DNA can easily occur (Cumpanas et al., 2020). Therefore, the use of strict negative controls and bioinformatic decontamination is essential to ensure data reliability. In addition, sampling strategies directly affect the representativeness of results. Studies have shown that the microbial composition of midstream urine differs from that of bladder urine obtained by catheterization or suprapubic aspiration, reflecting the predominance of the distal urethral microbiome and periurethral microbial communities. Rather than representing simple “contamination,” these differences are increasingly recognized as methodological and anatomical variations inherent to different sampling approaches (Bajic et al., 2020). Given the substantial methodological heterogeneity across urinary microbiome studies, full or partial standardization of the analytical workflow has become increasingly important for enabling cross-study comparison and clinical translation. Recent analyses highlight several critical steps where harmonization would yield meaningful improvements in data reproducibility (Isali et al., 2024): (i) urine collection method (clean-catch vs. catheterization), (ii) sample stabilization and storage, (iii) DNA extraction protocols optimized for low-biomass specimens, (iv) sequencing modality and library preparation, and (v) contamination-aware bioinformatic filtering. Synthesizing methodologies used across published studies, a generalized and reliable workflow would include standardized collection, low-biomass–appropriate extraction, parallel negative controls, quantitative biomass assessment, and validated filtering pipelines. Establishing such consensus-driven procedures will be essential for reducing protocol-driven variability and for advancing the urinary microbiome toward clinical applicability”.

**Figure 1:**
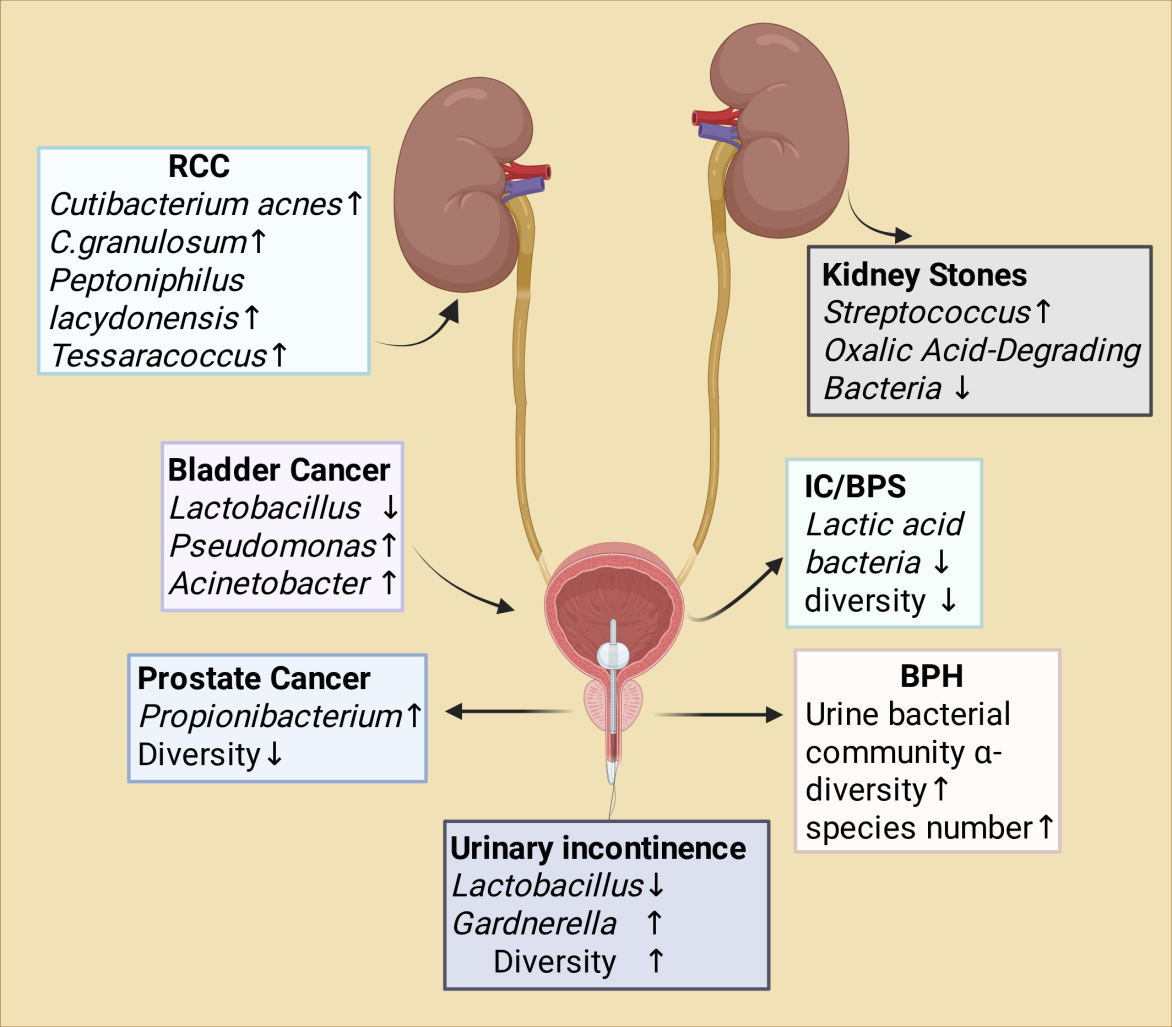
The relationship between urethral microorganisms and non-infectious urinary tract disorders. This schematic summarizes representative microbial signatures associated with different non-infectious urinary system diseases. Renal cell carcinoma (RCC) is characterized by increased abundance of Cutibacterium acnes, C. granulosum, Peptoniphilus lacydonensis, and Tessaracoccus. Bladder cancer is associated with reduced Lactobacillus and enrichment of Pseudomonas and Acinetobacter. Prostate cancer shows increased Propionibacterium and decreased microbial diversity. Interstitial cystitis/bladder pain syndrome (IC/BPS) is linked to reduced lactic acid–producing bacteria and overall diversity. Benign prostatic hyperplasia (BPH) is associated with increased urinary α-diversity and species richness. Urolithiasis is characterized by increased Streptococcus and reduced oxalate-degrading bacteria. Urinary incontinence is associated with decreased Lactobacillus, increased Gardnerella, and higher microbial diversity. Arrows indicate relative increases (↑) or decreases (↓) in abundance or diversity. RCC, Renal Cell Carcinoma. IC/BPS, Interstitial Cystitis/Bladder Pain Syndrome. BPH, Benign Prostatic Hyperplasia.

In summary, the advancement of urinary microbiome detection technologies has not only overturned the traditional concept of sterile urine but also opened new avenues for understanding the etiology and therapeutic strategies of urological diseases from a microecological perspective. **Figure 1** summarizes the evidence concerning the urinary microbiome and its association with non-infectious urinary tract disorders.

## Studies on the urinary microbiome in bladder cancer

Bladder cancer (BCa) is one of the most common malignancies of the urinary system. Chronic inflammation is considered one of its promoting factors, and resident urinary microbiota may contribute to bladder carcinogenesis by triggering persistent inflammation or producing bioactive metabolites (Alfano et al., 2016). Compared with healthy individuals, patients with bladder cancer exhibit a characteristic dysbiosis of the urinary microbiome, which, despite sharing an overall similar community structure, shows distinct compositional alterations that may be associated with tumor initiation, progression, therapeutic response, and diagnostic classification (Pallares-Mendez et al., 2024). Multiple studies have shown that, at the phylum level, the urinary microbiota of bladder cancer patients and healthy controls are broadly similar, being dominated by Bacillota, Proteobacteria, and Actinobacteriota. However, conclusions regarding alpha diversity (within-sample diversity) remain inconsistent: some studies found no significant difference (Bučević Popović et al., 2018; Hussein et al., 2021; Chorbińska et al., 2023), others reported markedly increased species richness and diversity in bladder cancer patients (Sheng et al., 2025), while still others observed reduced diversity (Chipollini et al., 2020). At more refined taxonomic levels, significant differences have been identified. For instance, the urine or tissue of bladder cancer patients often shows enrichment of genera such as Fusobacterium, Actinomyces, Acinetobacter, Pseudomonas, and Sphingomonas, whereas beneficial genera predominant in healthy urine, including Lactobacillus complex and Gardnerella, are relatively decreased (Bučević Popović et al., 2018; Wu et al., 2018; Bi et al., 2019; Sheng et al., 2025). These inconsistencies may stem from variations in study population characteristics such as sex composition and tumor subtype, as well as differences in sample size, collection methods, and sequencing techniques (Pederzoli et al., 2020; Chorbińska et al., 2023).

The characteristics of the urinary microbiome are closely associated with the aggressiveness and prognosis of bladder cancer. Studies have shown that patients with muscle-invasive bladder cancer (MIBC) have lower microbial diversity and richness compared with those with non-muscle-invasive bladder cancer (NMIBC), along with distinct compositional differences such as a higher abundance of Proteobacteria in the MIBC group. Specific genera, including Ralstonia and Propionibacterium, may serve as potential biomarkers for distinguishing tumor invasiveness (Sun et al., 2023). In addition, NMIBC patients with higher urinary microbial diversity after transurethral resection of bladder tumor tend to exhibit an increased risk of recurrence, suggesting that the urinary microbiome may serve as a prognostic indicator (Zeng et al., 2020). The potential of the urinary microbiome as a noninvasive biomarker is also attracting considerable attention. Studies employing large-scale 16S rRNA sequencing and machine learning have successfully developed diagnostic models, such as a random forest classifier based on 12 differential genera and a clinically applicable Patient Discrimination Index (PDI), both demonstrating strong discriminative performance with an area under the curve (AUC) of up to 89.1% (Sheng et al., 2025).Moreover, recent studies have classified urinary microbial communities into distinct subtypes, commonly referred to as “urotypes”, based on dominant taxa and clustering patterns. Such urotype-based frameworks have been reported in both healthy individuals and patients with bladder cancer, and may provide additional insights into disease heterogeneity and risk stratification (Gottschick et al., 2017; Sheng et al., 2025). These community patterns, such as Prevotella-dominant or Corynebacterium-dominant profiles, suggest that urinary microbial features may contribute to auxiliary diagnosis and personalized management. In terms of underlying mechanisms, the urinary microbiome may influence the progression of bladder cancer by modulating the tumor microenvironment. Certain bacteria can alter the cytokine milieu and matrix metalloproteinase activity of bladder wall cells, potentially promoting tumor invasion and metastasis. More importantly, the urinary microbiome may be associated with the immune response to tumor therapy. For instance, studies have found that patients with non-muscle-invasive bladder cancer (NMIBC) exhibiting high PD-L1 expression show greater microbial diversity, with enrichment of Fusobacteria and Ciliophora. This association may result from the induction of pro-inflammatory cytokines that upregulate PD-L1 expression, thereby facilitating immune evasion (Chen et al., 2022). These findings suggest that the urinary microbiome holds promise as a predictive biomarker for immunotherapeutic response. Furthermore, research has demonstrated a high degree of similarity between urinary and bladder tissue microbiota, along with notable sex-specific differences, providing a rationale for developing urine-based noninvasive diagnostic tools and gender-specific therapeutic strategies (Pederzoli et al., 2020).

In summary, current evidence indicates that patients with bladder cancer exhibit urinary microbiome dysbiosis. Specific microbial alterations, such as the enrichment of Fusobacterium and Acinetobacter and the depletion of Lactobacillus complex, are associated with tumor development, invasiveness, and therapeutic response. Although inconsistencies exist among different studies, the urinary microbiome undeniably represents a promising avenue for further investigation and clinical translation as a potential diagnostic biomarker, prognostic indicator, and therapeutic target in bladder cancer.

## Prostate cancer and the urinary microbiome

Prostate cancer (PCa) is the second most common malignancy among men worldwide (Bray et al., 2024). In recent years, increasing attention has been directed toward the role of the urethral microbiome in the development and progression of prostate cancer. Studies have demonstrated significant differences in the composition of urinary microbiota between prostate cancer patients and healthy individuals, and these differences appear to be more specific than those observed in fecal microbiota, suggesting that urinary microbes may serve as potential biomarkers for prostate cancer (Alanee et al., 2019). Comparative analyses of urinary microbiota across different genitourinary malignancies have revealed twelve bacterial species significantly enriched in the urine of prostate cancer patients, including Cutibacterium acnes and Cutibacterium granulosum. Notably, these bacteria are commonly elevated in both prostate and kidney cancer, whereas their abundance profiles differ from those seen in bladder cancer. Moreover, prostate cancer patients exhibit a lower Shannon diversity index of urinary microbiota compared with bladder cancer patients, indicating that tumor status may be associated with reduced microbial diversity (Ahn et al., 2022). Other studies have also reported an increased proportion of pro-inflammatory and opportunistic pathogenic bacteria in the urine of prostate cancer patients (Shrestha et al., 2018).

The microbiome may contribute to the pathogenesis of prostate cancer through multiple mechanisms. One of the key pathways involves chronic inflammation, in which microbial colonization of the lower urinary tract or prostatic ducts may activate inflammatory signaling cascades, inducing persistent low-grade inflammation and oxidative stress that lead to DNA damage and epithelial dysplasia (Sfanos et al., 2018). For example, Escherichia coli infection can cause chronic prostatitis and promote prostatic intraepithelial neoplasia (Simons et al., 2015), while Pseudomonas aeruginosa may enhance prostatic hyperplasia by activating the NF-κB pathway, thereby indirectly increasing the risk of malignant transformation (Li J. et al., 2024). In addition, microbial dysbiosis can modulate the tumor immune microenvironment by promoting macrophage polarization toward the tumor-supportive M2 phenotype (Liu et al., 2023). The gut microbiota also plays an important role through the gut–prostate axis, as its metabolites can influence androgen levels and immune function. Certain intestinal bacteria are capable of regulating androgen metabolism and may be implicated in the development of castration-resistant prostate cancer (Hays et al., 2024; Kim et al., 2024). In addition to these biological pathways, it is also important to consider the anatomical and physiological routes by which urinary microbes might influence prostatic tissue. It is unlikely that urinary microbes exert their effects on prostate cancer through direct luminal contact, as prostate cancer primarily arises in the peripheral zone, whereas direct exposure to urinary flow is anatomically limited to the transitional and periurethral zones. Current evidence instead suggests several indirect routes by which microbes detected in urine may influence carcinogenesis. These include hematogenous or lymphatic dissemination (Cohen et al., 2005), retrograde ascent through periurethral ducts (Shoskes et al., 2016), or inflammatory signaling originating in the transitional zone that subsequently affects the peripheral zone through interzonal stromal and immune networks (Sfanos et al., 2018). Such mechanisms align with findings of microbial DNA and inflammatory pathway activation within peripheral-zone prostate tissues, despite the absence of direct urinary contact.

Based on the above findings, the microbiome shows promising potential for clinical applications in prostate cancer. Urinary microbial profiles may serve as valuable tools for early screening and for distinguishing prostate cancer from benign prostatic hyperplasia (Cavarretta et al., 2017). Modulating the microbiota could also represent a novel strategy to enhance the efficacy of immunotherapy (Massari et al., 2019). Furthermore, variations in microbial composition may partly explain the racial and geographic disparities observed in prostate cancer incidence (Wang et al., 2025). In summary, prostate cancer is closely associated with the microbiome, particularly the urinary microbiome. Integrating microbial analysis into prostate cancer diagnosis and treatment in the future may provide new insights into the prevention and management of this heterogeneous disease.

## Renal cell carcinoma and the urinary tract microbiome

In recent years, studies on the urinary microbiome of renal cell carcinoma (RCC), including clear cell and papillary subtypes, have made preliminary progress. Evidence suggests that patients with RCC exhibit characteristic microbial dysbiosis, with specific bacterial taxa being either enriched or depleted in urine samples. A 16S rRNA sequencing–based study found that the urinary microbiota of RCC patients had lower alpha diversity, as measured by the Shannon index, compared with certain other genitourinary tumors, and that significant differences existed in beta diversity among groups. Compared with healthy individuals, RCC patients showed marked enrichment of Cutibacterium acnes, Cutibacterium granulosum, Peptoniphilus lacydonensis, and bacteria belonging to the genus Tessaracoccus (Ahn et al., 2022). Another prospective study reported that Gardnerella and Enterococcus were abundantly detected only in the urine of RCC patients, but were almost absent in those with benign renal cysts. Moreover, the preoperative urinary microbiota richness of RCC patients was significantly reduced and, although partially restored after surgery, remained lower than that of healthy controls (Cumpanas, 2024). These findings indicate a reconstructed urinary microbial community in RCC patients, suggesting a potential role of the urinary microbiome in the pathogenesis of renal cancer.

The microbiome contributes to the development of renal cancer through multiple mechanisms, with chronic inflammation and microbial metabolites playing pivotal roles. For instance, gut microbiota dysbiosis can disrupt tryptophan metabolism, leading to excessive production of kynurenine, which promotes renal cancer progression by activating the aryl hydrocarbon receptor (AhR) (Dai et al., 2021). Certain bacteria, such as Fusobacterium nucleatum, have also been associated with high PD-L1 expression, suggesting that microbes may facilitate immune evasion. Distinct microbial signatures have been observed across different renal cancer subtypes; for example, the expression of the succinate receptor (SUCNR1) correlates with specific microbial patterns and may serve as a prognostic indicator (Najm et al., 2022). These findings suggest that the urinary microbiome, both directly and through its interaction with gut microbial diversity, may be involved in the pathogenesis of renal cancer.

The urinary microbiome shows significant potential as a noninvasive biomarker. The detection of Gardnerella, Enterococcus, and microbial diversity indices may assist in the screening of clear cell renal cell carcinoma, while the higher abundance of Lactobacillus complex in papillary renal cell carcinoma provides clues for subtype differentiation. Microbial features may also serve as predictors of immunotherapy response, although current studies remain in the early stages. Future studies should expand sample sizes, refine sequencing technologies, and conduct longitudinal investigations to validate existing findings and further elucidate the role of microorganisms in the initiation and progression of renal cancer.

## Benign prostatic hyperplasia and the urinary microbiome

Benign prostatic hyperplasia (BPH) is a common cause of lower urinary tract symptoms (LUTS) in elderly men. Although its exact pathogenesis remains unclear, chronic prostatic inflammation is considered an important contributing factor, and dysbiosis of the urinary microbiome may influence the local immune microenvironment and play a role in disease progression (Oseni et al., 2023; Infante Hernández et al., 2024; Schifano et al., 2024). Current studies have shown that the alpha diversity of the urinary microbiota in BPH patients is significantly higher than that of individuals without BPH. A large-scale study involving 500 elderly men found that those with BPH had a greater number of microbial species in their urine, suggesting a positive correlation between microbial richness and the presence of BPH (Bowie et al., 2025). Moreover, patients with larger prostate volumes exhibited higher urinary microbial diversity, further supporting a link between microbiota composition and the degree of prostatic enlargement (Mariotti et al., 2024). Notably, animal studies have indicated that alterations in the beta diversity of gut microbiota are also associated with BPH, implying the existence of a gut–prostate axis and that microbial influence may extend beyond the urinary tract (Li et al., 2022). A study combining EQUC and 16S rRNA sequencing revealed that men with more severe LUTS, indicated by higher IPSS scores, were significantly more likely to test positive for bacteria in catheterized urine. For each one-point increase in IPSS, the odds of bacterial positivity increased by 2.21 times, providing the first clear evidence linking bladder urine microbiota to LUTS severity (Bajic et al., 2020). Another study comparing prostatic fluid microbiota between BPH and prostate cancer patients found that Eubacterium and Defluviicoccus were enriched in the BPH group, whereas members of the Lachnospiraceae family and the genus Propionicimonas were relatively reduced, suggesting that BPH possesses a distinct local microbial profile compared to prostate cancer (Yu et al., 2015).

The urinary microbiota may promote the progression of BPH by inducing and maintaining chronic inflammation of the prostate. Certain opportunistic pathogens, such as Escherichia coli, can colonize the urethra or bladder and trigger infiltration of inflammatory cells into prostatic tissue, along with the release of cytokines such as IL-6 and IL-8, which subsequently stimulate stromal cell proliferation (Ficarra et al., 2014). Histological inflammation is highly prevalent in BPH and has been associated with more severe symptoms and an increased risk of acute urinary retention (Alawamlh et al., 2018). Signaling pathways involving the NLRP3 inflammasome may be activated in this process, contributing to the formation of proliferative nodules and bladder outlet obstruction. Research on the relationship between BPH and the urinary microbiome remains at an early stage; however, growing evidence suggests that microbial dysbiosis may serve as a potential trigger for the development of BPH and the worsening of LUTS (Bechis et al., 2014). Future large-scale longitudinal studies are needed to clarify the causal relationships and to explore novel microbiota-targeted interventions, such as probiotics or microbiota transplantation, which may offer new therapeutic avenues for the prevention and treatment of BPH.

## Kidney stones and the urinary microbiome

Kidney stones are closely associated with the urinary microbiome, and their formation is influenced not only by classical infections caused by urease-producing bacteria but also by the overall regulation of the urinary and gut microbiota (Zhang et al., 2023). Traditionally, urease-positive bacteria such as Proteus and Klebsiella were believed to promote the formation of infection-related stones, such as struvite, by hydrolyzing urea and alkalinizing the urine. However, recent studies have shown that even in patients with non-infectious stones, such as calcium oxalate stones, the structure of the urinary microbiota is significantly altered. Recent findings further suggest that specific urinary bacteria may participate in early crystal–surface interactions, providing a mechanistic link between microbial presence and downstream processes such as crystal aggregation and biofilm-mediated growth (Noonin et al., 2024). Specifically, studies have demonstrated that bacteria can form biofilms on crystal surfaces, serving as cores or “nanostones” that facilitate crystal growth (Espinosa-Ortiz et al., 2019). The detection of bacterial DNA within some calcium oxalate stones further suggests that subclinical bacterial colonization may play a key role in initiating ostensibly sterile stone formation (Al et al., 2023; Lemberger et al., 2023). In addition, an integrated analysis of urinary microbiota and metabolomics revealed a significant enrichment of Streptococcus species in the urine of stone patients, along with 112 differential metabolites involving pathways such as unsaturated fatty acid and tryptophan metabolism. The correlation network between microbes and metabolites, including oxalate derivatives, was found to be restructured in patients with kidney stones, indicating that microorganisms may promote stone formation by modulating metabolic products (Gao et al., 2022).

Beyond the local bladder environment, systemic comorbidities appear to shape the urobiome of stone formers. A study on kidney stone patients with hypertension revealed that the co-occurrence of hypertension is associated with a distinct urinary microbial profile, characterized by an enrichment of Sphingomonas and alterations in nitrogen and nucleotide metabolic pathways (Liu et al., 2020a). This suggests that the urinary microbiome may reflect, or even contribute to, the complex interplay between metabolic syndrome and nephrolithiasis. Furthermore, recent advances have moved beyond bladder urine to investigate the upper urinary tract directly. By comparing bladder and renal pelvis urine collected after strict disinfection, Liu et al. confirmed the existence of a distinct renal pelvis microbiome. They found that while the pelvic and bladder microbiomes share similarities, the renal pelvis of stone formers exhibits specific alterations, such as a significantly higher abundance of Corynebacterium compared to non-stone controls (Liu et al., 2020b). These findings challenge the traditional reliance on voided urine and highlight the importance of site-specific sampling in understanding stone pathogenesis.

Research on the urinary microbiome has opened new avenues for the prevention and management of kidney stones. In the future, profiling urinary microbial characteristics may help assess the risk of stone recurrence, while microbiota-targeted interventions such as supplementing oxalate-degrading bacteria or inhibiting urease-producing bacteria may offer preventive strategies (Jung et al., 2023; Qiao et al., 2025). Some studies have already explored the use of engineered bacteria to degrade stone-forming components in urine, demonstrating promising therapeutic potential (Wan et al., 2024). However, before these approaches can be applied clinically, further research is needed to clarify the causal relationships between the microbiota and stone formation and to ensure their efficacy and safety.

## Interstitial cystitis or bladder pain syndrome and the urinary microbiome

Interstitial cystitis/bladder pain syndrome (IC/BPS) is a chronic condition characterized by persistent bladder pain accompanied by urinary frequency and urgency. Routine examinations typically reveal no clear evidence of infection, and the underlying pathogenesis remains incompletely understood (Vahlensieck, 2023). Recent studies have suggested that alterations in the urinary microbiome may play a role in the development and persistence of this disease. Multiple investigations have confirmed that the diversity and composition of urinary microbiota in IC/BPS patients differ significantly from those in healthy individuals. Abernethy and colleagues found that the species richness in bladder urine from IC patients was markedly reduced, with a significantly lower detection frequency of Lactobacillus complex, particularly Lactobacillus acidophilus, compared with healthy controls. The presence of Lactobacillus complex was also associated with lower symptom and pain scores (Abernethy et al., 2017). In addition, IC patients exhibited generally elevated levels of pro-inflammatory cytokines such as MDC and IL-4 in their urine, which were positively correlated with symptom severity, suggesting a close relationship between microbial dysbiosis and local inflammatory responses. Subsequent studies further supported that patients with IC/BPS often show a reduction in protective commensal bacteria such as Lactobacillus complex and an increase in opportunistic pathogens such as Sphingomonas, forming a characteristic state of microbiome imbalance (Zheng et al., 2023; Nandwana et al., 2024).

However, recent systematic reviews indicate that the microbial landscape in IC/BPS is more complex than a simple loss of diversity. While many studies report a depletion of Lactobacillus, others have observed no significant difference or even increased abundance in specific subgroups (Wolfe et al., 2023), highlighting high heterogeneity across studies (Fu et al., 2024). These discrepancies may partly reflect differences in sampling strategies, sequencing platforms, bioinformatic pipelines, patient selection criteria, and disease phenotyping. Emerging evidence also points to the involvement of non-bacterial components and metabolic interplay. A recent review highlighted that apart from bacterial dysbiosis, fungal species such as Candida and Saccharomyces are more prevalent during IC flares, suggesting a potential role for the mycobiome. Furthermore, viral agents like Epstein-Barr virus (EBV) and Polyomavirus have been detected in the bladder of IC/BPS patients, indicating a potential multi-kingdom etiology involving bacterial, fungal, and viral interactions. In terms of bacterial-immune interactions, the abundance of Sphingomonas has been positively correlated with urinary IL-6 levels, linking specific taxa to host inflammatory pathways (Nandwana et al., 2024). These findings underscore the need to look beyond bacterial taxonomy to functional interactions and multi-kingdom dynamics.

Although IC/BPS can be clinically classified into Hunner-type and non-Hunner-type forms, current studies have not identified significant differences in the overall structure of the urinary microbiota between the two. Both types exhibit a general trend of reduced Lactobacillus complex abundance and decreased microbial diversity (Nickel et al., 2019). Some reports suggest that male patients with the Hunner-type may display distinct microbial characteristics, but further data are needed to confirm these findings. Therefore, current microbiome research primarily highlights differences between IC/BPS patients and healthy individuals rather than distinctions among clinical subtypes. The underlying mechanisms may involve two main aspects. First, the loss of protective bacterial communities may render the urothelium more vulnerable to injury. A reduction in commensal species such as Lactobacillus complex could weaken the defense of the bladder mucosa, increasing susceptibility to low-grade bacterial irritation and promoting chronic inflammation (Hilt et al., 2014). Second, dysregulation of microbe–nerve–immune interactions may occur, where microbial imbalance activates mucosal immune responses and cytokine release, which in turn stimulate bladder nerves and lead to pain and urinary urgency (Scott et al., 2015). Another hypothesis proposes that some cases of IC/BPS may result from undetectable bacterial biofilms or intracellular bacterial communities within the bladder wall, causing persistent symptoms (Arya et al., 2012).

Analysis of the urinary microbiome has provided new insights into the diagnosis and treatment of IC/BPS. Specific microbial patterns, such as an increase in Gardnerella and a decrease in Lactobacillus complex, may serve as potential biomarkers to aid diagnosis. From a therapeutic perspective, several studies have explored strategies to modulate the urinary microbiota, including intravesical instillation of probiotics, oral administration of Lactobacillus complex preparations, and hypertonic glucose instillation. Some patients have shown symptomatic improvement accompanied by an increase in beneficial bacteria (Chen et al., 2025; Jhang et al., 2025). Although microbial alterations may represent only one component of the complex etiology of IC/BPS, interventions targeting the urinary microbiome hold promise as a novel direction for future treatment.

## Urinary incontinence and the urinary microbiome

The pathogenesis of urinary incontinence, including stress urinary incontinence (SUI) and urgency urinary incontinence (UUI), was traditionally considered unrelated to microbial factors. However, recent studies suggest that the urinary microbiome may play a role in the onset or exacerbation of symptoms. Multiple investigations have demonstrated significant differences in the urinary microbiota between patients with urinary incontinence and healthy individuals. Pearce and colleagues were the first to report that, compared with women without incontinence, those with UUI exhibited increased microbial diversity in bladder urine, a reduced proportion of Lactobacillus complex, and elevated abundances of potential pathogens such as Gardnerella, Prevotella, and anaerobic Streptococcus species (Pearce et al., 2014). Similarly, Komesu and colleagues found that among women under the age of 51, those with a moderate or mixed Lactobacillus profile in their urinary microbiota had a significantly higher risk of mixed urinary incontinence (MUI). In postmenopausal women, this association was weaker, suggesting that age may act as a modifying factor (Komesu et al., 2018). Furthermore, a multicenter study classified urinary microbiota into distinct community types and revealed that profiles characterized by low Lactobacillus complex abundance and high diversity were associated with more severe incontinence symptoms. In addition, urinary microbial richness was positively correlated with the frequency of leakage episodes (Carnes et al., 2024). Although most studies support a pattern of increased diversity and reduced Lactobacillus complex in patients with urinary incontinence, some reports have described decreased diversity in certain cases, possibly reflecting differences in study populations, hormonal status, or methodological approaches (Karstens et al., 2016). The prevailing mechanistic model proposes that under healthy conditions, the bladder is dominated by beneficial bacteria such as Lactobacillus complex, which help maintain microbial balance. When this equilibrium is disrupted, for instance due to declining estrogen levels, an increase in potential pathogens may lead to the production of irritant metabolites such as amines or trigger low-grade inflammation. These changes may enhance detrusor excitability or alter neural sensitivity, thereby contributing to the development of urgency and incontinence symptoms.

The association between urinary incontinence and dysbiosis of the urinary microbiota provides a new perspective for understanding the underlying mechanisms of this condition. In the future, specific microbial profiles may be used to predict surgical outcomes or therapeutic responses, thereby guiding individualized treatment strategies. At the same time, approaches such as intravesical instillation of probiotics or targeted modulation of the urinary microbiota to restore microbial balance may represent promising therapeutic options. However, the efficacy and safety of these interventions still require validation through large-scale randomized controlled trials.

## Association between microbiome alterations and disease mechanisms

Existing evidence indicates that dysregulation of the urinary microbiome plays a potential pathogenic role in various non-infectious urological disorders. To systematically elucidate these underlying connections, the following section summarizes the possible mechanisms through which microbial alterations may contribute to disease onset and progression, focusing on several common biological pathways. Although mechanistic insights from gut microbiome research are frequently used to contextualize urinary microbiome–disease interactions, the two ecosystems differ markedly in microbial biomass, nutrient availability, and mucosal architecture. As a result, disease-modifying mechanisms observed in the gut cannot be directly extrapolated to the urinary tract. Current evidence suggests that urinary microbes may be influenced by gut–urinary crosstalk through systemic immune modulation, metabolite circulation, or occasional translocation of microbial components, yet they also constitute a distinct low-biomass niche with unique host interactions (Miller et al., 2022; Noonin and Thongboonkerd, 2024; Yu D. et al., 2024). Therefore, the urinary microbiome should not be viewed as merely an extension of the gut microbiota; instead, its effects may operate through localized immune signaling and epithelial responses rather than the high-biomass and metabolically driven mechanisms characteristic of the gut.

### Chronic inflammation and immune regulation

Regulation of host immunity is one of the key functions of the microbiota. Although the aforementioned conditions are not typical infectious diseases, low-grade and persistent inflammation has been widely reported in disorders such as bladder cancer, benign prostatic hyperplasia or prostate cancer, and interstitial cystitis. Dysbiosis of the urinary microbiota may activate the innate immune responses of the bladder or prostate mucosa, for instance by engaging Toll-like receptors and triggering NF-κB signaling pathways, which lead to the release of pro-inflammatory cytokines (Lang et al., 2014). Specifically, repeated colonization of the prostate by Escherichia coli can induce chronic prostatitis, resulting in the accumulation of DNA damage and the development of precancerous lesions (Elkahwaji et al., 2009). Pseudomonas infection may similarly promote sustained prostatic hyperplasia through inflammatory responses. In IC/BPS, although traditional pathogens are not detected, tissue inflammation is still present and is thought to be driven by microbiota imbalance leading to sterile inflammation. The inflammatory microenvironment further exacerbates tissue injury, fibrosis, or tumor progression through the release of reactive oxygen species and various enzymes (Greten and Grivennikov, 2019). Therefore, the microbiota may contribute to disease by inducing a state of “microinflammation,” serving as an important bridge between infectious and noninfectious urological disorders.

### Microbial metabolites in stone formation and tumor metabolism

The microbiome is capable of synthesizing or degrading a variety of small-molecule metabolites, thereby altering the local physicochemical environment. For example, in kidney stone disease, both gut and urinary microbiota jointly regulate urinary oxalate levels. The absence of oxalate-degrading bacteria such as Oxalobacter can lead to hyperoxaluria and increase the risk of calcium oxalate stone formation (Sadaf et al., 2017), while urease-producing bacteria can raise urinary pH and promote phosphate crystallization (Subramaniyan et al., 2023). In the tumor microenvironment, microbial metabolic activity also plays an important role. Functional predictions of urinary microbiota in bladder cancer patients have shown enhanced pathways related to carbohydrate and nucleotide metabolism, which may reflect microbial contributions to the high metabolic demands of tumors (Sheng et al., 2025). Furthermore, metabolites produced by the gut microbiota, such as short-chain fatty acids and bile acids, can act on prostate tumor cells through systemic circulation, influencing their growth behavior (Yang and Kim, 2023). These findings suggest that microbial dysbiosis is often accompanied by alterations in metabolic profiles, which may contribute to disease pathogenesis by modulating epithelial cell metabolism and related signaling pathways.

### Direct microbial effects: toxins, enzymes, and genetic damage

Toxins, enzymes, and genetic damage: Certain bacterial products can directly act on host tissues and drive pathological processes. For instance, Escherichia coli produces the toxin colibactin, which induces double-strand DNA breaks in epithelial cells and is recognized as a classic genotoxic compound (Li et al., 2019). Although colibactin-producing strains have not yet been isolated from the prostate, this mechanism has been confirmed in other types of tumors. In addition, studies have shown that bacteria colonizing tumor tissues may influence the efficacy of radiotherapy and chemotherapy by altering local hypoxic conditions or modulating the activity of phagocytic cells (Routy et al., 2018).

**Figure 2:**
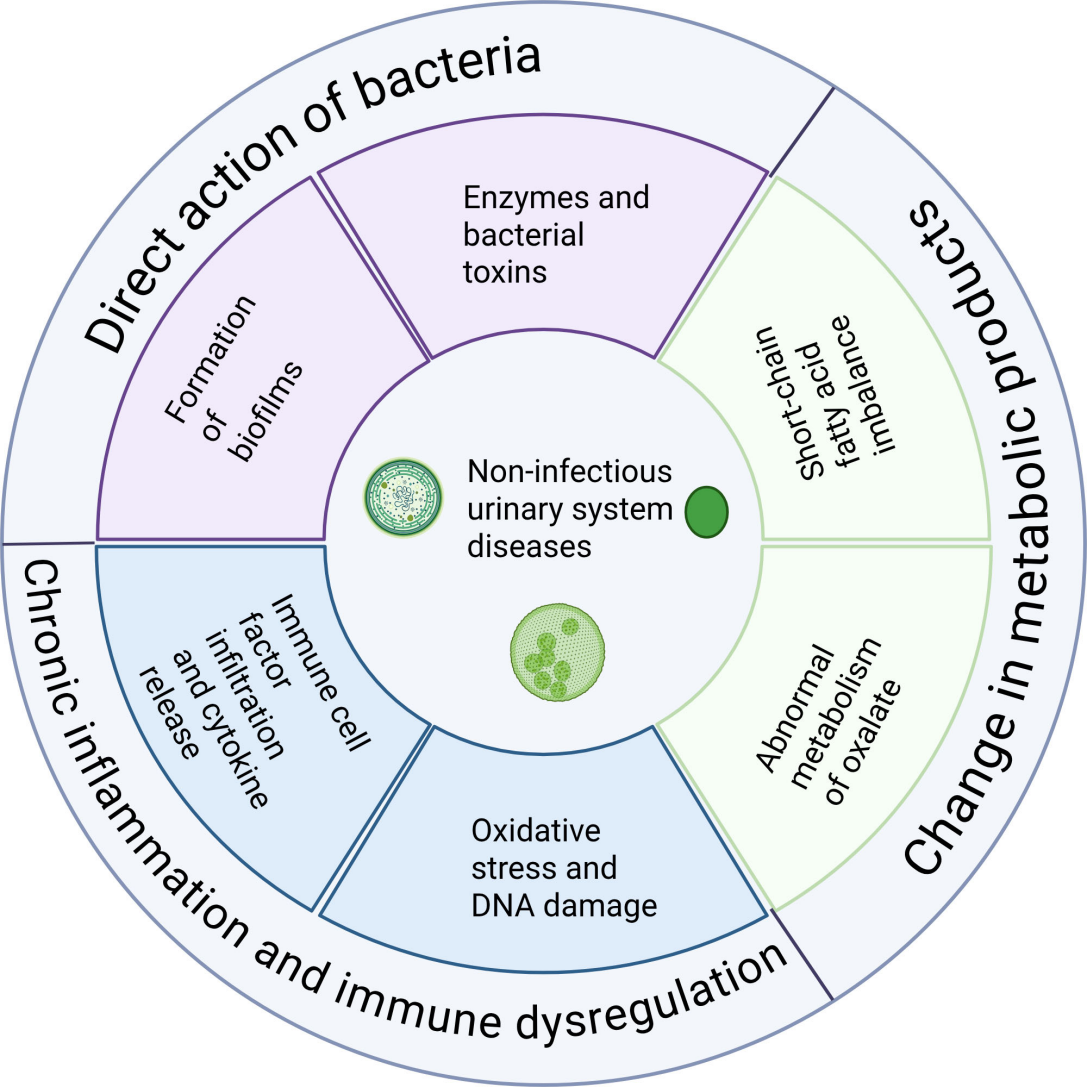
The core mechanism of the urinary microbiome in non-infectious urinary tract diseases. This schematic illustrates major biological pathways through which altered urinary microbial communities may contribute to disease pathogenesis. Direct bacterial effects include biofilm formation and the production of enzymes and toxins. Metabolic disturbances involve imbalances in short-chain fatty acids and abnormal oxalate metabolism. Dysbiosis may also promote chronic inflammation and immune dysregulation through immune cell infiltration and cytokine release, leading to oxidative stress and DNA damage. These interconnected mechanisms collectively contribute to the development and progression of non-infectious urinary system disorders.

In summary, the urinary microbiome contributes to the pathogenesis of various noninfectious urological diseases through multiple mechanisms, including modulation of inflammation and immunity, alteration of the metabolic environment, and direct interactions with epithelial tissues. **Figure 2** summarizes the key mechanistic roles of the urinary microbiome in noninfectious urological disorders. This framework helps explain why microbial dysbiosis is consistently observed across different diseases, as it may reflect shared underlying mechanisms involving inflammation and metabolic disturbance. Elucidating these processes in greater depth will not only enhance our understanding of disease pathophysiology but also provide a theoretical foundation for developing novel microbiota-based therapeutic strategies.

## The value of the microbiome in disease diagnosis, prediction, and classification

The potential of the urinary microbiome as a disease biomarker has attracted increasing attention. Because urine collection is relatively non-invasive and sequencing costs continue to decline, identifying specific microbial signatures associated with particular diseases could enable the development of diagnostic or predictive tools. Compared with traditional protein or metabolite biomarkers, urinary microbiome–based biomarkers offer several distinctive advantages. Microbial community signatures can reflect upstream ecological disturbances that occur earlier than downstream molecular changes, enabling potentially earlier disease detection. Unlike single-analyte biomarkers, microbiome profiles integrate host–microbe and microbe–microbe interactions, providing more comprehensive biological context. In addition, microbial patterns often remain stable across different sampling times and disease stages, thereby serving as robust indicators that complement existing molecular biomarkers. These features position microbiome-derived biomarkers as a valuable extension rather than a replacement of current diagnostic tools. Building on the evidence summarized in previous sections, the following highlights the potential applications of the urinary microbiome in disease diagnosis and classification.

### Early tumor screening and molecular subtyping

Microbial signatures identified in urine and tissue samples have provided new perspectives for the diagnosis and mechanistic understanding of urological malignancies. In bladder cancer, urinary microbiota display characteristic alterations, including a marked reduction in Lactobacillus complex and an increase in anaerobic genera such as Anaerococcus. Machine learning models based on these microbial markers have achieved diagnostic accuracies exceeding 80 percent (Sheng et al., 2025), demonstrating substantial potential for development into pre-screening diagnostic tools. Moreover, distinct microbial patterns may correspond to specific disease subtypes or risk levels. For example, enrichment of *Campylobacter* species in bladder cancer patients has been associated with disease severity (Kang et al., 2025), suggesting that microbial composition may serve as a basis for risk stratification. In prostate cancer, urinary enrichment of genera such as *Cutibacterium acnes* differentiates affected individuals from patients with other urological malignancies (Ahn et al., 2022). At the tissue level, the tumor microenvironment is characterized by reduced microbial diversity and an increased abundance of proinflammatory taxa, forming an “inflammatory microecology” (Cavarretta et al., 2017). These findings collectively establish the foundation for developing non-invasive diagnostic models based on urinary microbiota, while also highlighting the strong association between local microbial communities and the tumor immune microenvironment. Such associations suggest that urinary microbial profiling may serve as an indicator of tumor aggressiveness and help identify high-risk patients who may benefit from more intensive therapeutic interventions. In this context, microbial information can be leveraged for biological subtyping of urological cancers, offering a new dimension for precision oncology.

### Prediction of therapeutic response

The microbiome is increasingly being investigated for its potential to predict therapeutic efficacy. In bladder cancer, intravesical Bacillus Calmette Guerin (BCG) instillation remains a cornerstone immunotherapy for NMIBC, yet approximately one-third of patients fail to respond (Li R. et al., 2024). Previous studies have suggested that patients with higher urinary microbial diversity and enrichment of genera such as Leptotrichia tend to exhibit elevated PD-L1 expression and greater immune activity, which correlate with improved responses to BCG therapy (Chen et al., 2022). Therefore, profiling urinary microbiota prior to BCG treatment and analyzing the relationship between microbial composition and recurrence rates may help identify predictive microbial biomarkers of therapeutic response. Similarly, both gut and urinary microbiota may influence treatment outcomes in prostate cancer during endocrine or immunotherapy. While associations between gut microbiota and therapeutic efficacy have already been reported, the role of urinary microbiota remains to be elucidated and warrants further investigation. Identifying microbial features significantly associated with treatment response could enable pre-therapy stratification. For instance, a patient with a high abundance of Akkermansia in the gut may exhibit a better response to PD-1 inhibitor therapy, and such findings could be integrated into personalized treatment decision-making frameworks.

### Prediction of disease risk

For chronic urological conditions such as kidney stone disease and BPH, the microbiome holds great promise as a tool for predicting disease onset and progression risk. Studies have shown that individuals lacking colonization by Oxalobacter have a higher likelihood of developing calcium oxalate stones (Fargue et al., 2025). If validated and applied clinically, simple detection of Oxalobacter in stool or urine samples could serve as a screening method to identify high-risk individuals, who could then receive targeted dietary or probiotic interventions to reduce risk. In the context of BPH, if specific microbial taxa, such as certain anaerobic bacteria, are confirmed to accelerate disease progression or increase the risk of complications, microbial profiling could be incorporated into routine follow-up protocols for BPH patients. Correcting dysbiosis through microbial modulation might then become a preventive strategy to slow disease progression. For conditions with unclear etiology, such as IC/BPS, identifying early signs of microbial imbalance in asymptomatic high-risk populations could enable early warning and intervention, offering new opportunities for proactive disease management.

### Differential diagnosis

Microbiome profiling may also assist in differentiating between diseases with similar clinical presentations. For instance, prostate cancer and BPH often share overlapping symptoms, including elevated prostate-specific antigen (PSA) levels, which typically necessitate prostate biopsy for confirmation (Singh et al., 2025). Establishing discriminative microbial models based on urine or prostatic fluid composition could potentially help distinguish between benign hyperplasia and early malignancy. In this regard, recent studies utilizing metagenomic analysis of bacterial extracellular vesicles in urine have successfully differentiated prostate cancer from BPH at the phylum and class levels with promising diagnostic accuracy (Miyake et al., 2022). Similarly, conditions such as IC/BPS, overactive bladder (OAB), and chronic pelvic pain share overlapping symptoms but differ in etiology. In the future, microbial fingerprinting may assist in distinguishing among these syndromes. For example, IC/BPS is characterized by marked dysbiosis with a pronounced depletion of Lactobacillus complex, whereas patients with primary OAB may retain a stable Lactobacillus complex profile but exhibit transient microbial fluctuations (Yu WR. et al., 2024). Such microbial-based differentiation could facilitate more precise diagnosis and enable tailored therapeutic interventions.

It is important to emphasize that most studies investigating microbiome-based biomarkers are still at an exploratory stage, with limited sample sizes and a lack of validation for clinical application. The inherent variability of microbial communities and the substantial interindividual differences pose significant challenges to the development of universally applicable diagnostic models. Moreover, the clinical implementation of microbiome testing is currently constrained by issues related to cost, standardization, and methodological consistency. Nevertheless, with the continued advancement of sequencing technologies and bioinformatics, this field holds great promise. Once specific and stable microbial markers are identified for certain diseases, urinary microbiome analysis may become a routine component of health screening or clinical follow-up, providing non-invasive insights into disease risk and progression. It should also be noted that the existing literature on the urinary microbiome in non-infectious urological diseases is characterized by a pronounced geographic bias. Most available studies have been conducted in populations from high-income countries in the Global North, whereas data from low- and middle-income regions remain limited. This imbalance may restrict the generalizability of current conclusions, as urinary microbiome composition is influenced by ethnicity, dietary patterns, environmental exposures, socioeconomic status, and healthcare practices. Underrepresentation of diverse populations may obscure region-specific microbial signatures and disease associations. Future large-scale, multi-center, and multi-ethnic studies are therefore essential to establish globally applicable reference frameworks and to promote equity in urinary microbiome research.

## Current progress in microbiome-targeted interventions

Although most research on the urinary microbiome remains observational, emerging animal experiments and clinical trials are beginning to demonstrate the feasibility of microbiome-based interventions. In the field of nephrolithiasis, synthetic biology has offered a novel therapeutic strategy. A recent animal study utilized engineered Escherichia coli Nissle 1917 to express oxalate-degrading enzymes. In a rat model of hyperoxaluria, oral administration of these engineered bacteria significantly reduced urinary oxalate levels and inhibited calcium oxalate crystal formation (Wan et al., 2024), providing direct evidence that manipulating the microbiome can alter disease outcomes. In clinical settings, interventions targeting the bladder environment have shown promise in modulating the local microbiome. For instance, a recent study on Interstitial Cystitis/Bladder Pain Syndrome (IC/BPS) investigated the effects of intravesical dextrose prolotherapy. The results indicated that this metabolic intervention not only improved clinical symptoms but also reshaped the urinary microbiome by increasing the abundance of beneficial Lactobacillus and decreasing harmful genera such as Escherichia-Shigella (Chen et al., 2025). Furthermore, the use of probiotics, particularly Lactobacillus strains, is being explored to restore urinary homeostasis. While direct intravesical instillation remains experimental, oral probiotics have shown potential in reducing the recurrence of urinary tract infections and managing symptoms in BPH (Schifano et al., 2024), serving as a proof-of-concept for non-infectious urological diseases. Fecal Microbiota Transplantation (FMT) is another avenue, primarily targeting the gut-kidney axis. Case reports and early trials suggest that FMT may influence the efficacy of immunotherapy in urological cancers by reshaping the systemic immune environment (Routy et al., 2018).

However, as noted, the human body is a complex system. The success of these interventions depends not only on introducing beneficial microbes but also on overcoming “colonization resistance” and modulating the host immune response. Future clinical trials must rigorously assess the long-term safety and stability of these introduced communities.

## Conclusion

Research on the urinary microbiome is transitioning from descriptive association studies toward uncovering underlying mechanisms and achieving clinical translation. Future breakthroughs will depend on the standardization of sampling, sequencing, and analytical workflows, as well as on large-scale prospective cohorts and functional experiments that can validate microbial biomarkers, clarify causal relationships, and elucidate molecular mechanisms. At the same time, research should broaden its scope beyond bacteria to include fungi, viruses, and other components of the urinary microbiota, thereby achieving a more comprehensive understanding of the ecosystem. Ultimately, through deep interdisciplinary integration, these collective efforts are expected to drive the development of microbiome-based diagnostic tools and microecological therapeutic strategies, paving new avenues for the precise prevention, diagnosis, and treatment of urological diseases.
